# Amplifying Bioactivity of blue honeysuckle (*Lonicera caerulea* L.) fruit puree through Ultrasonication: Antioxidant and antiproliferative activity

**DOI:** 10.1016/j.ultsonch.2024.107179

**Published:** 2024-11-29

**Authors:** Wei Wu, Xiumei Ma, Yingqi Wang, Yating Yu, Junwei Huo, Dejian Huang, Xiaonan Sui, Yan Zhang

**Affiliations:** a Heilongjiang Green Food Science Research Institute, Northeast Agricultural University, Harbin 150030, PR China; b National-Local Joint Engineering Research Center for Development and Utilization of Small Fruits in Cold Regions, Northeast Agricultural University, Harbin 150030, PR China; c College of Horticulture and Landscape Architecture, Northeast Agricultural University, Harbin 150030, PR China; d Department of Food Science and Technology, National University of Singapore, 117543, Singapore; e College of Food Science, Northeast Agricultural University, Harbin 150030, PR China

**Keywords:** Blue honeysuckle, In vitro digestion, Polyphenols, Antiproliferative activity, LC-MS/MS

## Abstract

Blue honeysuckle (*Lonicera caerulea* L.) serves as a significant reservoir of polyphenol compounds. This impact of ultrasonication processing on the bioaccessibility of blue honeysuckle fruit puree during in vitro digestion was evaluated. The polyphenol compounds, antioxidant capacity and antiproliferative activity were measured, with a particular focus on determining the total proanthocyanidin content of the puree during digestion. The results revealed that the U300 W treatment significantly increased antioxidant content and enhanced the stability of antioxidant capacity, leading to stronger antiproliferative activity. A total of 33 compounds, including 14 phenolic acids, 5 flavanols, 1 flavanol-3-ol, 1 flavanone alcohol, 3 flavanones, 1 flavanone, and 8 non– polyphenols were found in both untreated and ultrasonicated puree during in vitro digestion. The untreated puree contained 22 compounds, while the ultrasonicated puree contained 33. Compared to untreated samples, ultrasonicated samples contained significantly higher levels of loganic acid, dihydrokaempferol, kaempferol derivatives, and plantagoside. Except for vanillic acid, citric acid, protocatechuic acid, and luteolin-4′-O-glucoside, the polyphenols showed a decreasing trend during oral-gastric-small intestinal-colon digestion. The U500 W ultrasonicated fruit puree exhibited the strongest antiproliferative activity. Overall, the results demonstrated that ultrasonication has the potential to enhance the bioaccessibility of antioxidant compounds and the antiproliferative activity of blue honeysuckle fruit puree.

## Graphical abstract

**Figure:**
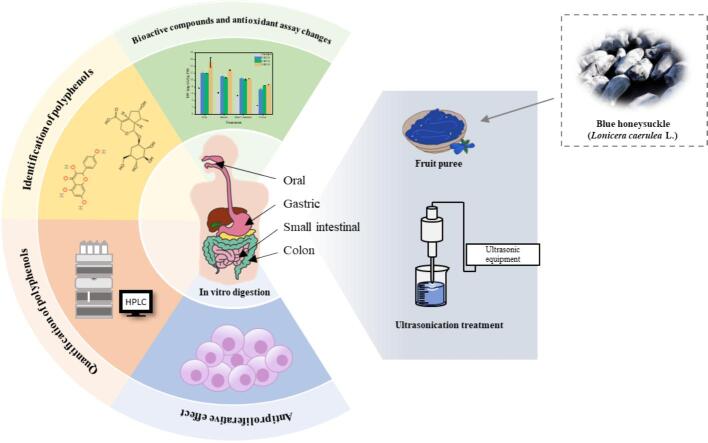

## 1 Introduction

Blue honeysuckles (*Lonicera caerulea* L.) are shrubs native to northern temperate regions. They are classified as a third-generation small berry. It is recognized for its dual role in medicine and food. This shrub thrives in cold regions of China, Russia, Japan, and North America, and serves as a significant reservoir of polyphenol compounds, which are naturally present in vegetables, fruits, and grains. Recent studies have highlighted blue honeysuckle's strong antioxidant, antidiabetic and other disease-fighting properties [1]. Zhang et al. comprehensively characterized and quantified polyphenols from 20 blue honeysuckle cultivars using HPLC-DAD and HPLC-ESI-QTOF-MS^2^ techniques. They identified 17 anthocyanins and 59 non-anthocyanin polyphenols in blue honeysuckle, with cyanidin-3-glucoside, myricetin-3-galactoside, and quercetin-3-galactoside being primary polyphenolic compounds [2]. Additionally, blue honeysuckle inhibits the proliferation of HCC cells by arresting the G2/M phase of the cell cycle. The findings showed that in HCC cells, blue honeysuckle extract inhibits proliferation both in vivo and in vitro by reducing the expression of _C_DC2 and cyclin B1, while enhancing the expression of MyT1 [3].

Plant polyphenols exhibit a wide range of physiological activities, but their ultimate usability by the human body depends on their bioavailability during digestion. Studying the bioavailability of polyphenols is crucial because biologically active substances must undergo a digestive process before they can affect the body, and this process can alter their structure and composition. Human digestion consists of two main phases: mechanical breakdown in the mouth and stomach, and enzymatic digestion in the small intestine. It has been shown that dietary polyphenols undergo hydrolysis of most glycosides in the small intestine, followed by methylation, sulfation, and glucuronidation [4]. Upon entering the colon, unabsorbed polyphenols are degraded into low molecular weight phenolic acids by gut bacteria [5]. In vitro digestion simulates the digestive process in a laboratory setting and is often used as an alternative to in vivo experiments. It offers advantages such as simplicity, speed, safety, and affordability compared to in vivo experiments. Bioaccessibility during digestion refers to the rates of compounds released from gastrointestinal matrix for absorption by the human [6]. It is an important and intuitive metric for assessing in vitro simulated digestion. The reports on the bioavailability of polyphenols from berries have been widely concerned. The stability and biological activity of polyphenols in wild blueberries and chokeberries during simulated in vitro digestion was analysed [7], [8]. Francesca et al. evaluated the phytochemical composition and antioxidant capacity of polyphenols from five berries (blackberry, blackcurrant, blueberry, raspberry, and strawberry) after in vitro gastrointestinal digestion. The results revealed that, although a large proportion of phenolics undergo transformation during digestion, berry polyphenols still exhibited antiproliferative effects, particularly those from blackcurrants [9]. Additionally, Mexican wild blackberries demonstrated notable antioxidant capacity and cell-based antioxidant capacity in Caco-2 cells after gastrointestinal digestion [10]. Ultrasonication, as a processing method, offers several advantages. Operating at lower temperatures compared to thermal processing, it helps preserve heat-sensitive nutrients and bioactive compounds, such as polyphenols, that are prone to degradation at high temperatures. Unlike thermal processing, ultrasonication is non-destructive, maintaining the original sensory qualities of the product [11]. Furthermore, ultrasonication consumes less energy and produces fewer greenhouse gases, making it an eco-friendly alternative. It also eliminates the need for chemical solvents [12]. Moreover, ultrasonication has bactericidal effects, which can enhance the shelf life of processed samples [13].

Blue honeysuckle is favoured by consumers for its natural, safe, green, and healthy properties. However, fresh fruit has a limited shelf life, so processing it into products can extend its shelf life. Ma et al. reported on the changes in physicochemical quality, sensory quality, and antioxidant properties of fermented goat milk with blue honeysuckle juice during in vitro digestion, finding that the addition of 4 % blue honeysuckle juice resulted in better quality [14]. For fresh fruit products, fruit puree is an excellent choice for infants, young children, and the elderly, as it requires no chemical additives. However, thermal processing can damage its nutrient content, especially anthocyanins. The purposes of this study were: (1) to determine the antioxidant contents and antioxidant capacity during in vitro digestion, evaluating the effect of ultrasound treatment on the absorption of blue honeysuckle puree in vitro. Notably, this includes the first-time determination of total proanthocyanidin content during in vitro digestion; (2) to identify the changes in polyphenol compounds during in vitro digestion, assessing the impact of ultrasound treatment on the absorption of polyphenols in vitro; and (3) to establish a theoretical foundation for the health benefits of ultrasound-treated blue honeysuckle puree. Future studies could investigate the effects of polyphenols in blue honeysuckle on cellular signalling pathways involved in antioxidant capacity and antiproliferative activity. Additionally, research could focus on the metabolic kinetics of these polyphenols in vivo, including their absorption, distribution, metabolism, and excretion.

## 2 Materials and methods

### 2.1 Materials

Methanol and 4-dimethylaminocinnamaldehyde (DMAC) were obtained from Tianjin Fuyu Fine Chemical Co. Ltd., China. Sodium hydroxide and ethanol were purchased from Tianjin Tianli Chemical Reagent Co. Ltd., China. Folin phenol reagent and TPTZ were supplied by Biotopped Science & Technology CO. Ltd. (Beijing, China). ABTS and DPPH were acquired from Bomei Biotechnology Co. Ltd. (Hefei, China). α-Amylase, pepsin, lipase, trypsin, and viscozyme L were sourced from Sigma-Aldrich Chemical Co., and bile was purchased from Shanghai Yuanye Biotechnology Co (Shanghai, Chian). Sodium carbonate, aluminium chloride, sodium acetate, sodium chloride, potassium chloride, and potassium persulfate were acquired from Kemio Chemical Reagent Co. Ltd. (Tianjin, China). The reagents used in the Caco-2 cells assays were prepared with cell-grade water.

Blue honeysuckle was harvested in June 2022 from the Xiangyang Farm of Northeast Agricultural University (126° 37′ 39′' E, 45° 42′ 22′' N). The blue honeysuckle used in this study is the cultivar ‘Lanjingling,’ cultivated and bred by the small berry group of Northeast Agricultural University.

### 2.2 Preparation of blue honeysuckle fruit puree

Blue honeysuckle fruit puree was prepared using a blender (JYL-Y912, Jiuyang Co., Ltd., Jinan, China). For thermal treatment, 50 mL of fruit puree was pasteurized in a water bath (DZKW-D-1, Tiantai Co., Ltd., Tianjin, China) at 85 °C for 15 min. For the ultrasonic treatment, each 50 mL of fruit puree was subjected to ultrasonication using an ultrasonic cell pulverizer (UX-600, Mitsui & Co., Ltd., Japan) at different power levels (100 W, 300 W, and 500 W) with a constant ultrasonic frequency of 20 kHz with 2 s intervals between pulses at 20 °C for 20 min. The sample temperature was maintained using a water bath. The untreated fruit puree served as the control. The samples were labelled as untreated, U100 W, U300 W, and U500 W, respectively.

### 2.3 In vitro gastrointestinal digestion

**Fig. 1:**
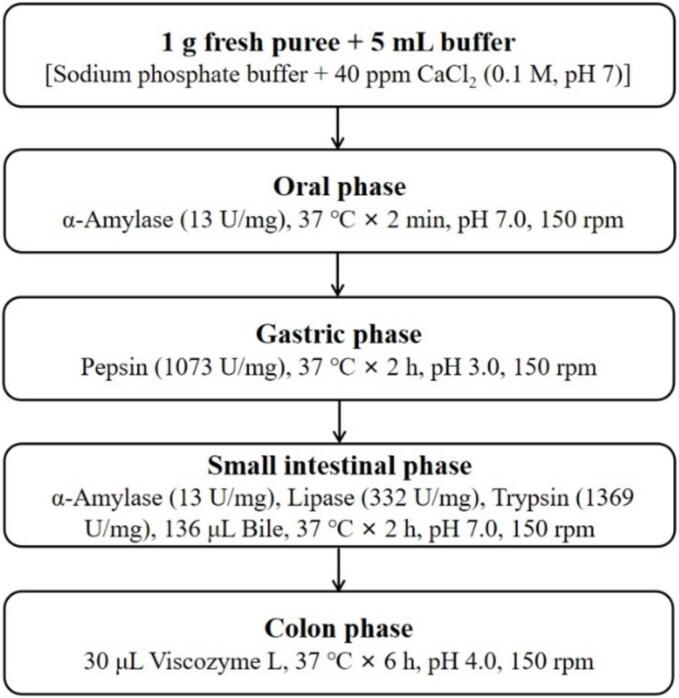
Overview and flow diagram of a simulated in vitro digestion.

Guo et al. described an in vitro gastrointestinal digestion model that emulates the physiological conditions of the oral, gastric, small intestinal, and colonic phases [15]. This procedure followed the enzyme concentrations outlined by Minkus et al. and utilized 6 M HCl and 0.9 M NaHCO_3_ for pH adjustments [16]. The blue honeysuckle fruit puree was combined with enzymes and maintained at 37 °C in a shaking water bath set to 150 rpm. The digestion process is illustrated in Fig. 1. For the oral digestion phase, 1 g of fresh puree was combined with 5 mL of sodium phosphate buffer (0.1 M, pH 7.0) containing 40 ppm CaCl_2_ and α-amylase (13 U/mg). The solution was then maintained at 37 °C for 2 min. For gastric digestion, the pH was lowered to 3.0, and the oral-digested sample was combined with pepsin (1073 U/mg) and incubated at 37 °C for 2 h. Intestinal digestion was performed in two stages: small intestine and colon. For the small intestine phase, the pH was adjusted to 7.0, and the gastric-digested sample was mixed with α-amylase (13 U/mg), lipase (332 U/mg), trypsin (1369 U/mg), and 136 µL of bile. This mixture was maintained at 37 °C for 2 h, after which the pH was adjusted to 4.0 for the colon phase, where 30 µL of viscozyme L was added, and the mixture was incubated at 37 °C for an additional 6 h to simulate the colonic environment. Supernatants from the oral, oral-gastric, oral-small intestinal, and oral-colon digestions were collected and centrifuged at 9000 rpm for 15 min.

### 2.4 Antioxidant contents

#### 2.4.1 Determination of total anthocyanin content (TAC)

TAC was traditionally measured using a microplate spectrophotometer (Epoch 2, Bio Tek Instruments, VT, USA) according to a modified version of the pH-differential method [17]. In this method, 180 µL of either buffer A (0.4 M sodium acetate, pH 4.5) or buffer B (0.025 M potassium chloride, pH 1.0) was combined with 20 µL of the sample in a 96-well plate. Absorbance readings were taken at 510 nm and 700 nm. The TAC in the digested blue honeysuckle fruit puree supernatants was calculated as milligrams of cyanidin-3-glucoside equivalent (C3GE) per liter using equations (1), (2).

(1) $$A={\left(A_{510}-A_{700}\right)}_{\mathrm{pH}=1.0}-{\left(A_{510}-A_{700}\right)}_{\mathrm{pH}=4.5}$$

(2) $$\mathrm{Total}\;anthocyanin\;content\;\left(mgC3GE/gFW\right)=\frac{A\times MW\times DF\times 1000}{\varepsilon \times L\times m}$$

where MW = 449.2 g/mol, the molecular weight of cyanidin-3-glucoside; DF represents the dilution factor; ε = 26,900 L mol^−1^ cm^−1^; L represents the optical path (1 cm); m represents the weight of the fresh fruit.

#### 2.4.2 Determination of total flavonoid content (TFC)

The determination of TFC was carried out using a spectrophotometric colorimetry method [18]. Twenty microliters of digested supernatants, deionized water and 5 % Na_2_CO_3_ solution were pipetted into a 96-well plate and maintained for 6 min. Twenty microliters of a 10 % AlCl_3_ solution was added, and the mixture was maintained for 10 min. A 4 % NaOH solution (40 µL) was then added and maintained for an additional 15 min. The absorbance of the reaction mixtures was monitored at 510 nm. The results were presented as milligrams of catechin equivalent (CE) per gram of fresh weight (FW).

#### 2.4.3 Determination of total phenolic content (TPC)

The TPC was measured using a modified version of the Folin-Ciocalteu method [19]. In brief, 20 µL of the digested sample was placed in a 96-well microplate, followed by 10 µL of Folin-Ciocalteu reagent and 90 µL of deionized water. After 5 min of dark incubation, 80 µL of a 75 g/L Na_2_CO_3_ solution was added, and the plate was maintained in the dark for 2 h. Absorbance was recorded at 765 nm. TPC was expressed as milligrams of gallic acid equivalent (GAE) per gram of fresh weight (FW).

#### 2.4.4 Determination of total proanthocyanidin content (TPAC)

TPAC was measured using the 4-dimethylaminocinnamaldehyde (DMAC) method with slight modifications as described by Adamson et al. [20]. A 4:1 ratio of 80 % ethanol to water was used to prepare the dilution solution. Acidified ethanol was prepared by mixing ethanol, deionized water, and hydrochloric acid in a 75:12.5:12.5 vol ratio (v/v/v). DMAC solution was created by dissolving DMAC in the acidified ethanol. Seventy microliters of the sample were pipetted into a 96-well plate, followed by the DMAC solution. Absorbance was recorded at 640 nm over a 30-minute period. The results were expressed as milligrams of procyanidin A2 equivalents (PA2E) per gram of fresh weight (FW).

### 2.5 Determination of antioxidant capacity of blue honeysuckle

#### 2.5.1 DPPH radical scavenging assay

The antioxidant capacity of the digested samples was assessed using the DPPH assay, based on the method outlined by Brand-Williams et al. with minor adjustments [21]. In brief, 5 µL of the digested sample was mixed with 195 µL of a 25 mg/L DPPH solution and maintained in the dark for 2 h. Absorbance was then determined at 515 nm. The antioxidant activity was calculated using a standard equation, and the results were expressed as milligrams of Trolox equivalents (TE) per gram of fresh weight (FW).

(3) $$DPPH\;\left(\%\;inhibition\right)=\left(1-\frac{A_{i}}{A_{0}}\right)\times \;100$$

where A_0_ represents the absorbance values of blank and A_i_ represents the absorbance value of samples.

#### 2.5.2 ABTS radical scavenging assay

To conduct the ABTS assay, an ABTS^•+^ solution was prepared by combining 10 mL of 7 mM ABTS with 10 mL of 2.45 mM K_2_S_2_O_8_ in water, following the method of Re et al. [22]. This mixture was maintained in the darkness at 20 °C for 12 h to develop the ABTS^•+^ radicals. The assay involved mixing the digested sample with the ABTS^•+^ solution, and after 6 min, the absorbance was measured at 734 nm. The ABTS radical scavenging activity of the blue honeysuckle digestion sample was calculated and expressed as milligrams of Trolox equivalents (TE) per gram of fresh weight (FW).

(4) $$ABTS\;\left(\%\;inhibition\right)=\left(1-\frac{A_{i}}{A_{0}}\right)\times \;100$$

where A_0_ represents the absorbance values of blank and A_i_ represents the absorbance value of the samples.

#### 2.5.3 Ferric reducing antioxidant power (FRAP)

The FRAP assay for the digested sample solution was conducted based on the method outlined by Benzie & Strain [23]. To prepare the FRAP reagent, TPTZ (dissolved in HCl), FeCl_3_·6H_2_O, and an acetate buffer (pH 3.6) were combined in a 1:1:10 vol ratio. The reagent was heated to 37 °C, and its absorbance at 593 nm was used as the blank measurement. The digested sample and deionized water were mixed with the FRAP reagent, and after 30 min, the absorbance was again measured at 593 nm. The antioxidant capacity of the blue honeysuckle digestion sample was expressed as milligrams of Fe^2+^ equivalent per gram of fresh weight (FW), calculated using equation (5).

(5) $$FRAP\;\left(\%\;inhibition\right)=\left(1-\frac{A_{i}}{A_{0}}\right)\times \;100$$

where A_i_ is the absorbance of the sample; and A_0_ is the absorbance of the blank.

### 2.6 The absorption of bioactive compounds during digestion

The absorption rates of polyphenols were calculated using equation (6). The results were expressed as percentages.

(6) $$Absorption\;rate\;\left(\%\right)=\left(1-\frac{A_{i}}{A_{0}}\right)\times \;100$$

where A_i_ is the value of bioactive compounds contents or antioxidant radical assay of polyphenols during one digestion phase; and A_0_ is the value of that during the previous digestion phase.

### 2.7 Cell line and culture conditions

Caco-2 cells, a human colorectal adenocarcinoma line, were maintained in Modified Eagle's Medium (MEM) enriched with fetal bovine serum (10 %) and penicillin–streptomycin (1 %). The cells were maintained under humidified conditions at 37 °C with a 5 % CO_2_ atmosphere.

### 2.8 Evaluation of the cell proliferation

For the in vitro experiment, Caco-2 cells were plated in 96-well plates and allowed to acclimate for 72 h before anti-proliferative treatments were applied. Cells in the logarithmic growth phase were counted after digestion with 0.25 % trypsin. To estimate the number of viable and metabolically active cells, the microculture tetrazolium test (MTT) was employed. MTT solution was added to each well, and the cells were maintained at 37 °C for 4 h in a 5 % CO_2_ environment. Mitochondrial dehydrogenases reduce tetrazolium salt into purple formazan crystals in direct proportion to cell viability. Following incubation, the supernatant was removed, carefully avoiding the crystals. DMSO was then added to dissolve the formazan, and the plate was shaken for 10 min. Absorbance was measured at 490 nm using an ELISA plate reader. Cell viability, indicating the anti-proliferative effect, was calculated using equation (7).

(7) $$Y\;\left(\%\;inhibition\right)=\left(\frac{A_{i}-A_{0}}{A}\right)\times \;100$$

where Y is cell viability rate, A_i_ is the absorbance value of samples at 490 nm, A_0_ is the absorbance value of the zeroing hole, A is the difference which is the absorbance value of the samples at 490 nm minus the control group zeroing hole.

### 2.9 Identification and quantification of polyphenols using HPLC-ESI-QTOF-MS^2^

In this study, non-anthocyanin polyphenols were detected using high-performance liquid chromatography with diode-array detection (HPLC-DAD, 1260 series, Agilent, USA) and high-performance liquid chromatography with electrospray ionization quadrupole time-of-flight mass spectrometry (HPLC-ESI-QTOF-MS^2^, AB Sciex, CA, USA). Polyphenols were separated using a C18 column (Luna 5 μm, 250 mm × 4.6 mm, Phenomenex, CA, USA) and detected at 280 nm. The mobile phase consisted of two components: A (20 % 50 mM ammonium acetate in water, 80 % acetonitrile, pH 3.6) and B (200 mM formic acid, pH 2.6). The gradient elution started at 14 % A and 86 % B, progressing to 16.5 % A and 83.5 % B at 12.5 min, 25 % A and 75 % B at 17.5 min, and 80 % A and 20 % B by 40 min. The injection volume was 10 μL, with a flow rate of 1.0 mL/min, and the column was maintained at 25 °C. For mass spectrometry, the instrument was set to perform full scans over *m*/*z* 100–2000 (MS^1^) and *m*/*z* 50–2000 (MS^2^) in negative ionization mode. The ion spray voltage, declustering potential, collision energy, and collision energy spread were set to −4500 V, −100 V, −40 V, and 20 V, respectively. The nitrogen carrier gas flow rate was maintained at 35 mL/min, with source gas and curtain gas pressures set at 55 psi and 35 psi, respectively. The source temperature was regulated at 550 °C.

### 2.10 Statistical analysis

The data from HPLC-ESI-QTOF-MS2 were processed using PeakView 2.0 software. Calculations and analysis were carried out with Microsoft Excel 2016 (Office 2016, Microsoft, Redmond, WA), while statistical evaluations were performed using one-way ANOVA followed by Duncan's least significant difference test in SPSS Statistics 20.0 software. Graphical representations were generated with Origin 2019 (OriginLab, Massachusetts, USA). Results were reported as mean ± standard deviation, with significance established at P < 0.05.

## 3 Results and discussion

### 3.1 Total phenolic content (TPC) of different ultrasonic blue honeysuckle fruit puree

**Fig. 2:**
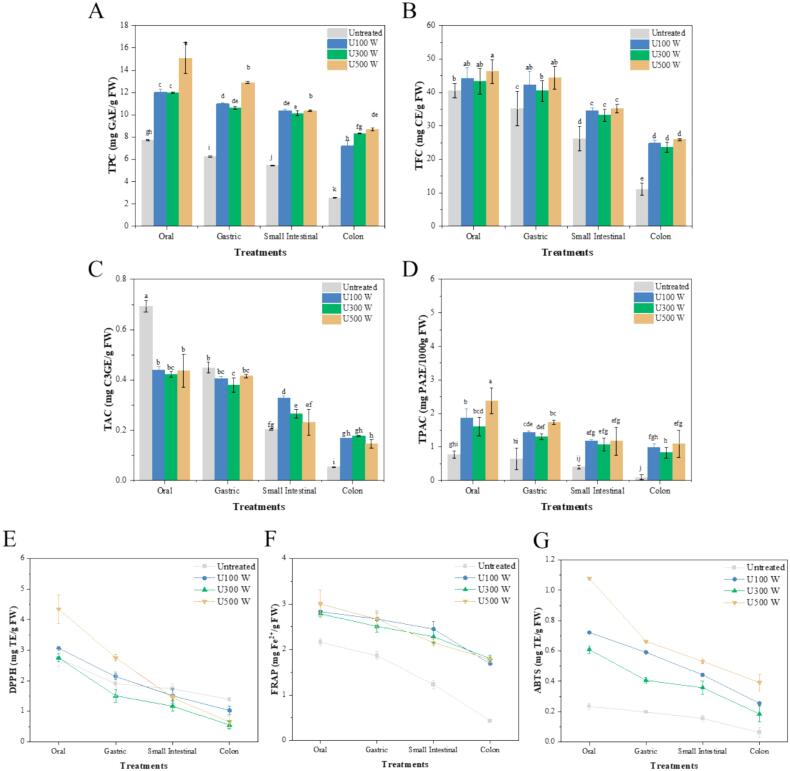
Changes in antioxidant contents and antioxidant capacity of in vitro digested blue honeysuckle puree. (A) The total phenolic (mg GAE/g FW); (B) The total flavonoid (mg CE/g FW); (C) The total anthocyanin (mg C3GE/g FW); (D) The total proanthocyanidin (mg PA2E/g FW); (E) DPPH (mg TE/g FW); (F) FRAP (mg Fe^2+^/g FW); (G) ABTS (mg TE/g FW). (For interpretation of the references to colour in this figure legend, the reader is referred to the web version of this article.)

We determined the TPC of blue honeysuckle puree to evaluate the effect of different ultrasonic power levels during in vitro digestion. The TPC of various treated fruit purees is summarized in Fig. 2. As shown in Fig. 2, the TPC of ultrasonic-treated blue honeysuckle fruit puree significantly (P < 0.05) increased compared to the untreated fruit puree. Specifically, the TPC of U500 W fruit puree showed a significant increase compared to U100 W and U300 W fruit purees during oral digestion, reaching up to 15.09 ± 1.40 mg GAE/g FW. It was observed that the digestion samples of ultrasonic-treated puree had higher TPC than the untreated puree during oral digestion. The observed increase may result from the ultrasonic treatment breaking down the cell walls of blue honeysuckle fruit, thereby releasing more polyphenol compounds. Previous studies have shown that ultrasonic cavitation energy enhances the release of polyphenols by disrupting their bonds with polysaccharides and cell wall proteins, facilitating their extraction from intracellular compartments [24]. Wang et al. reported that ultrasonic-treated noni (*Morinda citrifolia* L.) juice had a 2.67 % higher TPC than untreated juice, demonstrating a similar increase in phenolic content due to ultrasonication [25].

Previous research has seldom focused on the in vitro digestion of ultrasonic-treated fruit puree. In our study, we observed that the TPC of samples decreased during whole in vitro digestion, from oral to gastric, small intestinal, and colon digestion stages. Specifically, the TPC of U100 W and U300 W treatments decreased the most during colon digestion, by 30.54 % and 17.75 %, respectively. In contrast, the TPC of the U500 W treatment decreased the most (19.77 %) during small intestinal digestion. The pH of the small intestinal environment was set to 7.0 for simulating gastrointestinal digestion, a slightly neutral environment. During small intestinal digestion, apart from the natural processes of digestion and absorption, the reduction in TPC may primarily be attributed to changes in the pH level of the digestive environment. The poor bioavailability of anthocyanins can be partially attributed to their instability when exposed to the mildly alkaline conditions present in the small intestine [26]. From oral to colon digestion, the TPC of U100 W, U300 W, and U500 W treated fruit purees decreased by 40.06 %, 30.33 %, and 42.48 %, respectively, which were lower than the TPC decrease of untreated fruit puree (67.01 %). The results of this study suggest that the improvement in the bioavailability of blue honeysuckle fruit puree may be attributed to ultrasonic treatment.

### 3.2 Total anthocyanin content (TAC) of different ultrasonic blue honeysuckle fruit puree

As shown in Fig. 2, we summarized the changes in TAC of different ultrasonic-treated blue honeysuckle fruit purees during in vitro digestion. During oral digestion, the TAC of the untreated puree sample was 0.693 ± 0.023 mg C3GE/g FW, which was notably higher (P < 0.05) compared to the samples treated with ultrasound. The reduced levels of anthocyanins in the ultrasound-treated samples may be attributed to severe physical conditions created within bubbles during cavitational collapse, as well as to various simultaneous or isolated sonochemical reactions. This degradation may be attributed to the sonolysis of water, resulting from cavitation and the subsequent generation of hydroxyl radicals. These radicals can cause the chemical decomposition of anthocyanins by opening ring structures and converting them into chalcones, likely due to the elevated temperatures produced by sonication [27].

The TAC of blue honeysuckle fruit puree declined during digestion. Notably, the TAC of untreated, U100 W, and U300 W blue honeysuckle fruit puree decreased the most during colon digestion, by 73.89 %, 48.78 %, and 33.21 %, respectively. In contrast, the TAC of U500 W blue honeysuckle fruit puree decreased the most during small intestinal digestion, by 44.34 %. During gastric digestion, the TAC of both untreated and ultrasonic-treated blue honeysuckle fruit purees decreased the least. Specifically, the TAC of ultrasonic-treated (U100 W, U300 W, and U500 W) blue honeysuckle fruit puree decreased by 7.52 %, 9.95 %, and 5.03 %, respectively, which was notably lower than the 35.21 % decrease observed in untreated blue honeysuckle fruit puree. The enhanced stability of anthocyanins during gastric digestion is likely due to the acidic environment (pH 3.0), which favors the formation of the more stable flavylium cation. Research on blueberries and red cabbage has demonstrated that anthocyanins remain notably stable in the acidic conditions typical of the stomach [28]. Ultrasonication at 500 W may increase the sensitivity of anthocyanins to α-amylase, lipase, and trypsin, resulting in greater reductions during small intestine digestion, while other treatments caused more significant TAC reductions during colon digestion. The notable reduction can be linked to structural modifications of anthocyanins at neutral pH (7.0), leading to the hydration of these pigments and yielding colorless carbinol pseudobases, which subsequently convert to colorless chalcones. Interestingly, although the initial TAC of untreated blue honeysuckle fruit puree (0.693 ± 0.023 mg C3GE/g FW) was significantly higher than that of ultrasonic-treated blue honeysuckle fruit puree, by the end of colon digestion, the TAC of untreated blue honeysuckle fruit puree (0.053 ± 0.002 mg C3GE/g FW) was significantly lower than the ultrasonic-treated samples. From oral to gastric, small intestinal, and colon digestion, the TAC of untreated blue honeysuckle fruit puree decreased the most, by 92.35 %, compared to ultrasonic-treated blue honeysuckle fruit puree. This indicates that ultrasonication could improve the bioavailability of blue honeysuckle fruit puree.

### 3.3 Total flavonoid content (TFC) of different ultrasonic blue honeysuckle fruit puree

Fig. 2 shows the TFC of digestion samples throughout in vitro digestion. Across all digestion stages-oral, gastric, small intestinal, and colonic-the TFC in samples treated with ultrasound was significantly greater (P < 0.05) compared to untreated samples. During the oral digestion phase, the TFC in ultrasound-treated samples was notably higher than in untreated samples. Specifically, the TFC of the sample treated at 500 W (46.31 ± 3.56 mg CE/g FW) was 1.14 times greater than that of the untreated sample (40.58 ± 2.16 mg CE/g FW). The highest degradation rate of TFC occurred during colonic digestion. This may be due to the phenomenon of “C-ring fission” of flavonoids, where the C-ring (the central ring in the structure of flavonoids) is enzymatically degraded during digestion, forming smaller structures [29]. Different digestive enzymes play various roles, making flavonoid structures unstable under optimal pH environments. As a result, the TFC gradually decreases during digestion and absorption.

### 3.4 Total proanthocyanidin content (TPAC) of different ultrasonic blue honeysuckle fruit puree

Berries are among the best sources of proanthocyanidins, a crucial component of polyphenols. Proanthocyanidins are often described as “dual-purpose molecules” because of their wide range of health benefits [30]. Understanding how ultrasonic treatment influences the digestion of proanthocyanidins is essential. As shown in Fig. 2, the TPAC in the ultrasonic-treated samples was notably higher compared to the untreated ones. Compared with TPC, TAC and TFC, TPAC also gradually decreases due to digestion and absorption. The TPAC of untreated samples decreased the most. Among the ultrasonic treatments, the U500 W treatment (2.37 ± 0.38 mg PA2E/g FW) had significantly higher TPAC than the U100 W (1.86 ± 0.29 mg PA2E/g FW) and U300 W (1.60 ± 0.27 mg PA2E/g FW) treatments during oral digestion. Different ultrasonic treatments showed varying absorptivity during the four stages of digestion. The U500 W treatment exhibited better absorption during gastric (27.01 %) and small intestinal digestion (32.37 %). The U300 W treatment showed better absorption (23.15 %) during colon digestion. Overall, the U500 W treatment demonstrated the best absorption (54.01 %) throughout the entire digestion process. Higher ultrasonic power led to better TPAC absorption. These changes in TPAC may be attributed to cavitation caused by ultrasonication, which affects various chemical, physical, and biological reactions [31]. The localized high temperatures and pressures generated by ultrasonication can weaken the chemical bonds of phenolic compounds, making them more prone to degradation [11]. Additionally, ultrasonication reduces particle size in fruit puree, increasing the surface area of solid particles. This enhanced surface area facilitates greater interaction with digestive enzymes, improving the dissolution and absorption of proanthocyanidins. Ultrasonication also impacts the activity of endogenous enzymes within fruit purees [12], which may further promote the release of proanthocyanidins in in vitro digestion models.

### 3.5 Antioxidant capacity of blue honeysuckle fruit puree

Polyphenols are natural antioxidants with diverse structures, resulting in varying antioxidant capacity. These compounds significantly impact human health by mitigating the threat of many diseases. To evaluate the antioxidant capacity of the digestion samples we conducted three radical scavenging assays: ABTS, DPPH, and FRAP. The results are shown in Fig. 2 E-G.

The antioxidant capacity of the untreated sample showed the lowest decrease in DPPH and the highest decrease in ABTS and FRAP assays. For DPPH, the U500 W treatment had significantly (P < 0.05) higher values than other ultrasonic treatments during oral digestion. Because polyphenols are digested and absorbed in various digestive environments, ultrasonic treatment showed the highest absorption during colon digestion, with U500 W treatments showing the highest DPPH absorption. Regarding ABTS, the U500 W treatment exhibited the highest antioxidant radical scavenging activity, significantly outperforming other ultrasonic treatments during digestion. The ABTS value of the U500 W treatment decreased by 63.76 %. For the FRAP assay, ultrasonic treatments showed significantly higher values compared to untreated samples during colon digestion. Notably, the U300 W treatment exhibited the best stability in antioxidant radical scavenging, with a reduction rate of 35.25 %. The observed decline in antioxidant capacity can be attributed to the degradation of TPC during in vitro digestion. Research indicates that polyphenols undergo degradation, polymerization, and oxidation throughout the digestive process. The antioxidant efficacy of these compounds is influenced by their structural characteristics, including the number and arrangement of hydroxyl groups and the nature of substitutions on aromatic rings. TPC in digestion samples decreases progressively as they are absorbed by the body. Similar degradation or transformation of antioxidants has been noted during the digestion of fruit purees, such as persimmons [32]. These findings suggest that ultrasonic treatment enhances the stability of TPC during digestion. A positive relationship between TPC and antioxidant capacity has been documented in other studies, such as those involving prickly wines [33]. Therefore, ultrasonic treatment resulted in a more stable antioxidant capacity during digestion.

Different ultrasonic treatments exhibited varying rates of decline during digestion. It was observed that the DPPH of the U500 W treatment was significantly (P < 0.05) absorbed during colon digestion. The ABTS of the U300 W treatment was absorbed the most during gastric digestion, and the FRAP of the U100 W treatment showed the highest absorption during colon digestion. The sharp decline in antioxidant radical assays during colonic digestion suggests that most antioxidants may be degraded at this stage, similar to findings in previous studies on pomegranate peels [34].

### 3.6 Identification of polyphenol compounds in digestion samples

**Fig. 3:**
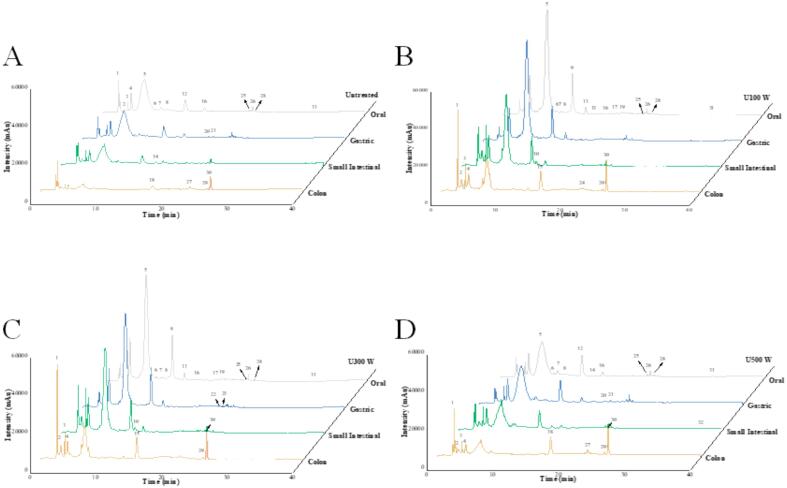
HPLC chromatogram (UV 280 nm) of phenolics from different treatments of blue honeysuckle puree during in vitro digestion. (A) Untreated; (B) U100 W; (C) U300 W; (D) U500 W. (For interpretation of the references to colour in this figure legend, the reader is referred to the web version of this article.)

To investigate the specific changes in polyphenols during the digestion of blue honeysuckle puree, the composition of polyphenol compounds after in vitro digestion, including the oral, gastric, small intestinal, and colon samples, was analysed using HPLC-ESI-QTOF-MS^2^. Table 1 details the MS data for non-anthocyanin polyphenols, while Table S1 provides the quantitative measurements for these compounds. Fig. 3 summarizes the HPLC chromatogram at 280 nm for non-anthocyanin polyphenols during in vitro digestion. In total, 33 compounds were identified in the digestion samples, comprising 14 phenolic acids and their derivatives, 5 flavonols and derivatives, 1 flavanol-3-ol, 1 flavanone alcohol, 3 flavanones, 1 flavanone, and 8 additional non-phenolic substances.

**Table 1:** Detection and identification by HPLC-ESI-QTOF-MS^2^ of bioactive compounds in blue honeysuckle puree from different treatments during in vitro digestion.

Peak	RT (min)	Chemical formula	MW	Exact mass (*m*/*z*)	MS (*m*/*z*) [M – H]^-^	MS^2^ (*m*/*z*)	Error (ppm)	Tentative identification	λ max (nm)	Reference
**Phenolic acids and derivatives**										
1	2.38	C_14_H_8_O_9_	330	328.8613	328.8615	118.9416/260.8727	0.135	Vanillic acid glucose	322	[38]
2	3.34	C_6_H_8_O_7_	192	191.0204	191.0204	111.0085/173.0094/191.0199	−0.0511	Citric acid	265	[39]
5	6.94	C_16_H_23_O_10_	376	375.1285	375.1285	213.0767	0.0715	Loganic acid	282	[40]
9	10.51	C1_6_H_18_O_9_	354	353.0892	353.0891	179.0367/191.0578	−0.1781	3-Caffeoylquinic acid	320	[38]
10	11.22	C_16_H_18_O_9_	354	353.0888	353.0887	173.0456/179.0366/191.0551	−0.4979	4-Caffeoylquinic acid	317	[38]
12	12.47	C_16_H_18_O_9_	354	353.0872	353.0872	173.0457/191.0568	0.0383	5-Caffeoylquinic acid	320	[38]
15	15.37	C_9_H_8_O_4_	180	179.0348	179.0349	135.0447/179.0360	0.4315	Caffeic acid	317	[38]
17	17.17	C1_6_H_18_O_8_	338		337.0935	163.0409/191.0570		Coumaroylquinic acid	242,283	[36]
19	17.97	C_18_H_34_O_5_	330	329.089	329.0888	167.0360/168.3497/191.0361/221.0462/239.0595	−0.5939	Vanillic acid hexoside	282	[39]
21	19.77	C_14_H_18_O_9_	330	329.0875	329.0878	209.0458	0.3763	Vanillic acid-4-glucoside	284	[35]
22	20.87	C_7_H_6_O_4_	154	153.0207	153.0207	109.0291	0.0988	Protocatechuic acid	300	[39]
24	21.73	C_9_H_8_O_3_	164		163.039	119.0493		Coumaric acid	306	[36]
27	22.81	C_7_H_6_O_4_	154	153.0202	153.0203	109.0297/153.0190	0.4239	Protocatechuic acid	281	[39]
**Flavonols and derivatives**										
3	3.65	C_15_H_12_O_6_	288	287.0914	287.0914	125.0261	0.2509	Dihydrokaempferol	284	[39]
4	4.24	C_31_H_35_O_16_	664	663.1318	663.1315	285.0405	−0.3163	Kaempferol derivative	281	[39]
23	21.15	C_26_H_28_O_16_	596	595.1336	595.1343	151.0034/178.9995/301.0367/447.0952	1.2477	Quercetin-hexoside-pentoside	288	[39]
26	22.75	C_27_H_30_O_16_	610	609.1446	609.1442	301.0358	−0.7875	Quercetin-3-O-rutinoside	286	[35]
33	37.45	C_27_H_30_O_15_	594	593.1309	593.1306	285.1036/593.1326	−0.4991	Kaempferol-3-O-rutinoside	245	[36]
**Flavan-3-ol**										
25	22.37	C_22_H_18_O_10_	442	441.1757	441.1758	169.0853	0.3538	Epicatechin gallate	429	[39]
**Flavanonol**										
16	15.51	C_21_H_22_O_12_	466	465.103	465.1031	303.0517	0.2458	Taxifolin-3-O-hexoside	279	[35]
**Flavanones**										
11	12.36	C_21_H_24_O_10_	466	465.1046	465.1045	125.0257/151.0365/285.0423/303.0539	−0.3331	Plantagoside	340	[41]
14	14.17	C_27_H_32_O_16_	612	611.1609	611.1616557	611.1623	0.7943	Eriodictyol-O-dihexoside	296	[36]
18	17.23	C_27_H_35_O_13_	568	567.1926	567.1925	328.8675	−0.1647	Dimethoxylflavanone derivative	283	[42]
**Flavone**										
13	13.78	C_21_H_20_O_11_	448	447.1517	447.1517	174.9550/269.1032	−0.1121	Luteolin-4′-O-glucoside	340	[39]
**Other compounds**										
6	8.24	C_27_H_35_O_13_	568	567.191	567.1908	521.1879	−0.1916	Saccharide	220	[37]
7	8.68	C_21_H_24_O_10_	436	435.1496	435.1498	273.0407	0.127	Phloretin-2′-O-glucoside	236,327	[43]
8	9.92	C_28_H_22_O_6_	454		452.9224	252.5791		Cis-ε-viniferin	271	[41]
20	19.18	C_26_H_29_O_15_	582	581.2076	581.208	246.9458/248.9591/521.1872/535.2046	0.4018	Gentiabavaroside	277	[40]
28	23.05	C_28_H_24_O_7_	472	471.1879	471.1878	121.0665	−0.1273	Restrisol A	249,332	[41]
30	25.87		210		209.0805	141.0909/209.0794		Unknown	265	
31	32.30	C_18_H_26_O_10_	402	401.0856	401.0854	269.0825	−0.1856	Benzyl alcohol-hexoside- pentoside I	246	[35]
32	36.77		252		251.0957	120.9605/251.0970		Unknown	358

#### 3.6.1 Phenolic acids and derivatives

Fourteen phenolic acids and their derivatives were detected in the fruit puree digest. Peak 1 showed an anionic *m*/*z* of 329 [M−H]^-^ and a fragment ion at *m*/*z* 119 and 261, tentatively identified as vanillic acid glucose. Peak 2 yielded an *m*/*z* of 191 [M−H]^-^ and a fragment ion at *m*/*z* 111, attributed to the ion [M−H−COOH−COOH−H−H]^-^. It was tentatively assigned as citric acid. Peak 5 showed an anionic *m*/*z* of 375 [M−H]^-^, calculated for C_16_H_23_O_10_ (loganic acid), with a fragment ion at *m*/*z* 213, may dropping a glucose. Peaks 9, 10, and 12 shared the same parent ion *m*/*z* of 353 [M−H]^-^ and yielded the fragments at *m*/*z* 179 and 191. These peaks were tentatively characterized as caffeoylquinic acid isomers: 3-caffeoylquinic acid, 4-caffeoylquinic acid, and 5-caffeoylquinic acid, respectively. Peak 19 showed an anionic *m*/*z* of 329 [M−H]^-^ with a fragment at *m*/*z* 167 [M−H−162]^-^, corresponding to vanillic acid, with the 162 Da mass loss due to hexoside moiety. The peak was tentatively characterized as vanillic acid hexoside. Peaks 22 and 27, despite different retention times suggesting diverse structures, showed the same molecular anion at *m*/*z* 153 [M−H]^-^, with a fragment at *m*/*z* 109, tentatively assigned as protocatechuic acid.

#### 3.6.2 Flavonols and derivatives

Two quercetin (fragment at *m*/*z* 301) derivatives and three kaempferol (fragment at *m*/*z* 285) derivatives were detected in the fruit puree digest. Peaks 4 and 33 both exhibited the characteristic kaempferol fragment ion at *m*/*z* 285, but the parent ions were different at *m*/*z* 663 [M−H]^-^ and 594 [M−H]^-^, respectively. Therefore, peak 4 was tentatively identified as a kaempferol derivative, and peak 33 as kaempferol-3-O-rutinoside. Peak 23 ([M−H]^-^, *m*/*z* 595) and peak 26 ([M−H]^-^, 609) both yielded fragment ions at *m*/*z* 301 ([M−H−294]^-^) and 301 ([M−H−308]^-^), due to the loss of hexoside-pentoside and rutinoside, respectively. Consequently, they were tentatively characterised as quercetin-hexoside-pentoside and quercetin-3-O-rutinoside [35].

#### 3.6.3 Flavan-3-ol

Peak 25 produced an anionic *m*/*z* 441 [M−H]^–^, which fragmented into *m*/*z* 169. With a chemical formula determined as C_22_H_18_O_10_, peak 25 was tentatively characterized as epicatechin gallate.

#### 3.6.4 Flavanonol

Peak 16 showed an anionic *m*/*z* 465 [M−H]^–^, which fragmented into *m*/*z* 303 [taxifolin]^–^ with the loss of a hexoside moiety (162 Da). Its chemical formula was determined to be C_21_H_22_O_12_. Therefore, peak 16 was tentatively characterized as taxifolin-3-O-hexoside [35].

#### 3.6.5 Flavanones

Peak 11 yielded an *m*/*z* of 465 [M−H]^–^ which fragmented into *m*/*z* 125, 151, 285, and 303. It was tentatively identified as plantagoside. By comparing the ion fragments with literature data, peaks 14 and 18 were tentatively characterized as eriodictyol-O-dihexoside and dimethoxylfla vanone derivative, respectively [36].

#### 3.6.6 Flavone

Peak 13 produced an ion at *m*/*z* 447 [M−H]^-^, with a loss of 162 Da, indicating a glycoside, and was identified as lignoceroside-4′-O-glucoside.

#### 3.6.7 Other compounds

Peak 6 yielded an anionic *m*/*z* at 567, fragmenting into *m*/*z* 521, and was tentatively determined as a saccharide [37]. Based on previous studies, peaks 8, 28, and 31 were identified as *cis*-ε-viniferin, restrisol A, and benzylalcohol-hexoside-pentoside I, respectively. Peak 30 produced an anionic *m*/*z* at 209 [M−H]^-^ with fragments at *m*/*z* 141 and 209. Peak 32 showed an anionic *m*/*z* at 251 [M−H]^-^, with fragments at *m*/*z* 120 and 251. Both peaks were tentatively unknown.

### 3.7 Characterization and quantitative analysis of polyphenol compounds in digestion samples

**Fig. 4:**
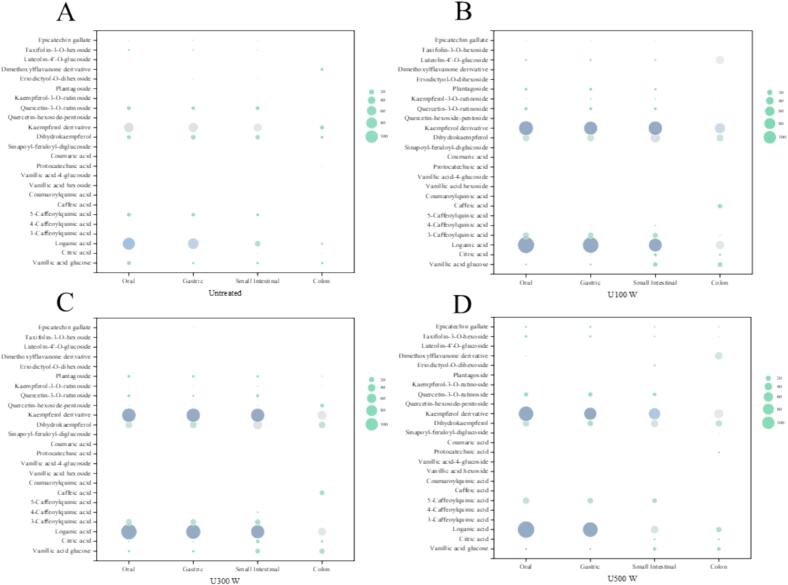
Detection of changes of polyphenols content of in vitro digested blue honeysuckle puree by HPLC-ESI-QTOF-MS2. (A) Untreated; (B) U100 W; (C) U300 W; (D) U500 W. (For interpretation of the references to colour in this figure legend, the reader is referred to the web version of this article.)

During digestion, we identified and quantified polyphenol compounds, marking the first instance of such comprehensive analysis. The results, summarized in Table S1, detail the changes in polyphenol contents during simulated in vitro digestion. The HPLC chromatograms of digestion samples are presented in Fig. 4.

Untreated samples revealed 22 polyphenol compounds during digestion. Ultrasonic treatments identified 22 (U100 W), 23 (U300 W), and 22 (U500 W) polyphenol compounds, respectively, during digestion. Previous studies have reported that ultrasonication can accelerate the release of polyphenols from intracellular structures [24]. Ultrasonication notably increased the presence of seven types of phenolic acids and derivatives during digestion, with U300 W treatment being particularly effective. Notably, only U300 W treatment produced protocatechuic acid, which increased significantly (P < 0.05) as digestion progressed. Similarly, only U100 W treatment produced coumaric acid (1.0755 ± 0.0188 mg/100 g FW) during colon digestion. Among the phenolic acids and derivatives, loganic acid had the highest content (20.7042 ± 0.7548 mg/100 g FW − 166.7610 ± 0.8622 mg/100 g FW), and ultrasonic treatments significantly (P < 0.05) enhanced its levels. Except for vanillic acid glucose, citric acid, and protocatechuic acid, other phenolic acids and derivatives decreased during digestion. It's suggested that polyphenols may transform into new small molecules during colon digestion [44]. The content of vanillic acid glucose (17.6303 ± 0.3075 mg/100 g FW) increased the most during the entire digestion process under U100 W treatment. Citric acid showed the highest content during small intestinal digestion, with U300 W treatment exhibiting the highest levels (9.5254 ± 0.0474 mg/100 g FW). Regarding flavonols and their derivatives, kaempferol derivative was the compound with the highest content, followed by dihydrokaempferol. Ultrasonic treatment generally increased the content of these compounds, except for quercetin-3-O-rutinoside, which was completely absorbed during colon digestion. Quercetin-hexoside-pentoside was exclusively found in U300 W treatment and increased during digestion. As for flavanones, flavone, and flavanonol, ultrasonication engendered plantagoside and luteolin-4′-O-glucoside and increased the content of taxifolin-3-O-hexoside of U500 W significantly (P < 0.05). Additionally, ultrasonication enhanced the content and absorption of epicatechin gallate during digestion. As for other compounds, ultrasonic treatment improved the absorption of phloretin-2′-O-glucoside and retrisol A.

During gastric and small intestinal digestion, the content of phenolic acids and flavonoids decreases. Goulas and Hadjisolomou reported a significant loss of polyphenol compounds during small intestinal digestion [45]. However, for certain polyphenols, such as vanillic acid, protocatechuic acid, and luteolin-4′-O-glucoside, which not only resisted degradation but increased during digestion. It is probable that their absorption is facilitated by the activity of polysaccharide-complex enzymes and other digestive enzymes. This process may also lead to the formation of new substances. This increase can be attributed to changes in pH, enzymatic reactions, and the presence of other molecules in the matrix [46]. During colon digestion, most polyphenols were converted into small molecules, and a new phenolic acid, coumaric acid, was produced, as reported by Gowd et al. [44].

### 3.8 Anti-proliferative activity of blue honeysuckle fruit puree with different treatments after colon digestion

**Fig. 5:**
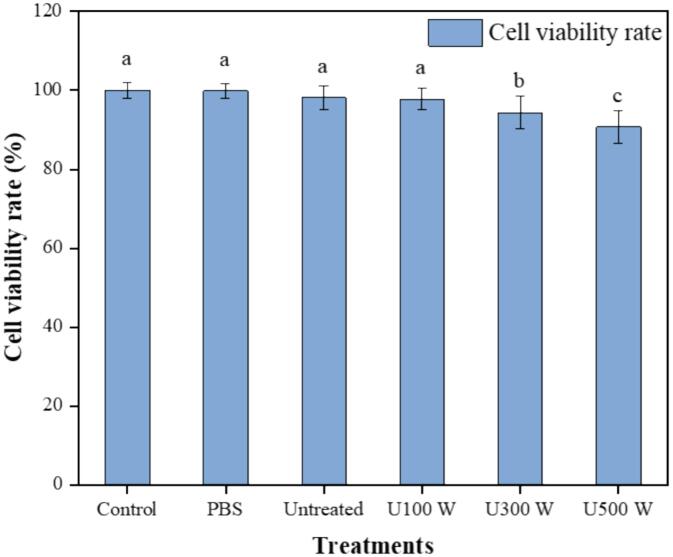
Effect of different treatments of colonic digest of blue honeysuckle puree on Caco2 cells. (For interpretation of the references to colour in this figure legend, the reader is referred to the web version of this article.)

Cancer remains a major global health issue, significantly contributing to morbidity and mortality rates. Anthocyanins, the primary antioxidants found in blue honeysuckle, provide a range of health benefits. These include potent antioxidant effects, anti-inflammatory properties, and potential anti-cancer activity. Cheng et al. reported that blue honeysuckle fruit exhibits potent anticancer activity [1]. In this study, we determined the antiproliferative activity of ultrasonic-treated digestion samples for the first time, assessing their effects on Caco-2 cell viability. Lower cell viability indicates stronger antiproliferative activity. As shown in Fig. 5, the survival rates of Caco-2 cells in the puree from U300 W and U500 W treatments were 94.41 % and 90.75 %, respectively, considerably less effective compared to the other treatments (P < 0.05). Furthermore, the cell survival rate for the U500 W treatment was notably lower compared to the U300 W treatment. This greater inhibition of Caco-2 cell proliferation could be attributed to the higher concentration of bioactive compounds, such as polyphenols from blue honeysuckle. Chen et al. have previously highlighted a significant link between antiproliferative effects and total polyphenol content in oats [47]. Polyphenols may exert a cytotoxic effect on neoplastic cells by acting as pro-oxidants. Previous studies have shown that polyphenols can interfere with the dual role of reactive oxygen species (ROS). As secondary messengers in intracellular signalling cascades, ROS can contribute to the induction and maintenance of the oncogenic phenotype in cancer cells. However, ROS can also induce cellular senescence and apoptosis, functioning as anti-tumorigenic agents. By disrupting cellular redox balance, ROS can enhance cytotoxic activity, inhibiting cell proliferation [48]. While in vitro antiproliferative activity does not account for the uptake and metabolism of bioactive compounds, these results provide valuable insights for designing future preclinical studies on the anticancer effects of blue honeysuckle in colon cancer.

## 4 Conclusion

In this study, blue honeysuckle puree was processed using ultrasound, a non-thermal processing method. The effects of ultrasonication during in vitro digestion were also investigated. This research marks the first time the TPAC of ultrasonicated samples was determined during in vitro digestion. The results revealed that ultrasonication increased TPC, TAC, and TFC of the digestion samples, thereby enhancing their antioxidant capacity and antiproliferative activity. The U300 W treatment exhibited the highest stability in antioxidant capacity and the greatest diversity of polyphenol compounds. However, the U500 W treatment demonstrated superior absorption of antioxidant compounds and antiproliferative activity. In conclusion, ultrasonication, as a non-thermal processing, is advantageous for enhancing the absorption of polyphenols in blue honeysuckle fruit puree during in vitro digestion. However, it is vital to note that simulated in vitro digestion has some limitations and requires further exploratory studies.

## CRediT authorship contribution statement

**Wei Wu:** Writing – original draft. **Xiumei Ma:** Data curation. **Yingqi Wang:** Formal analysis. **Yating Yu:** Methodology. **Junwei Huo:** Resources. **Dejian Huang:** Validation. **Xiaonan Sui:** Visualization, Validation. **Yan Zhang:** Writing – review & editing, Supervision, Conceptualization.

## Declaration of competing interest

The authors declare that they have no known competing financial interests or personal relationships that could have appeared to influence the work reported in this paper.
