# Supplementary material for: Amplifying Bioactivity of blue honeysuckle (Lonicera caerulea L.) fruit puree through Ultrasonication: Antioxidant and antiproliferative activity
Source: Ultrason Sonochem. 2024 Nov 29;112:107179. doi: 10.1016/j.ultsonch.2024.107179 (PMC11647649; doi:10.1016/j.ultsonch.2024.107179)
Supplement: Supplementary Data 1 [file mmc1.docx]

**Supplementary material**

**Tabe S1.** Detection and identification of the bioactive compound content of in vitro digested blue honeysuckle puree from different treatments by HPLC-ESI-QTOF-MS².

**Supplementary material**

**Table S1.** Detection and identification of the bioactive compound content of in vitro digested blue honeysuckle puree from different treatments by HPLC-ESI-QTOF-MS². Different capital letters in the table indicate statistically significant differences between the contents of different substances in the same class (P < 0.05). Different lowercase letters in the table indicate significant differences (P < 0.05) in the content of the same substance among different treatments.

| Peak | Compound | Content (mg/100 g) | | | | | | | | | | | | | | | |
| --- | --- | --- | --- | --- | --- | --- | --- | --- | --- | --- | --- | --- | --- | --- | --- | --- | --- |
|  |  | U-O | U-G | U-S | U-C | U100 W-O | U100 W-G | U100W-S | U100 W-C | U300 W-O | U300 W-G | U300 W-S | U300 W-C | U500 W-O | U500 W-G | U500 W-S | U500 W-C |
| **Phenolic acids and derivatives** | | | | | | | | | | | | | | | | | |
| 1 | Vanillic acid glucose | 10.8753±0.0963^Be^ | 5.5371±0.0302^Ch^ | 5.8692±0.1131^Cg^ | 5.0773±0.1461^Aij^ | 3.7089±0.0141^Cl^ | 4.3567±0.0137^Ck^ | 14.8947±0.0657^Cd^ | 17.6303±0.3075^Cb^ | 5.0094±0.0170^Cj^ | 6.0185±0.0190^Cg^ | 16.8522±0.0839^Cc^ | 19.4252±0.2717^Ba^ | 5.3171±0.0275^Chi^ | 3.7761±0.0212^Cl^ | 10.0733±0.2039^Cf^ | 10.6879±0.3211^Be^ |
| 2 | Citric acid | 0.2095±0.0019^Cn^ | 0.3462±0.0019^Dm^ | 1.3182±0.0254^Dk^ | 1.2698±0.0365^Dk^ | 1.5150±0.0058^Dj^ | 1.6917±0.0053^Di^ | 8.6286±0.0381^Bb^ | 5.2002±0.0907^Dd^ | 2.4379±0.0083^Dg^ | 2.0968±0.0066^Dh^ | 9.5254±0.0474^Da^ | 5.7016±0.0797^Dc^ | 0.9962±0.0052^Dl^ | 0.9668±0.0054^Dl^ | 4.2295±0.0856^De^ | 4.0331±0.1470^Cf^ |
| 5 | Loganic acid | 94.4056±0.8357^Ai^ | 74.7180±0.4080^Aj^ | 21.3718±0.4118^An^ | 4.3101±0.1240^Bo^ | 159.1760±0.6070^Ab^ | 148.7594±0.4674^Ac^ | 104.3649±0.4603^Ah^ | 44.7145±0.7800^Ak^ | 144.7364±0.4924^Ad^ | 126.9040±0.4007^Af^ | 110.5968±0.5505^Ag^ | 41.9227±0.5864^Al^ | 166.7610±0.8622^Aa^ | 135.4395±0.7611^Ae^ | 37.7824±0.7648^Am^ | 20.7042±0.7548^An^ |
| 9 | 3-Caffeoylquinic acid | - | - | - | - | 27.4634±0.0503^Ba^ | 22.2191±0.0770^Bc^ | 18.9137±0.1730^Be^ | 1.8167±0.2335^Ef^ | 27.4473±0.2335^Ba^ | 23.5567±0.4170^Bb^ | 21.1243±0.3002^Bd^ | 0.5818±0.0174^Eg^ | - | - | - | - |
| 10 | 4-Caffeoylquinic acid | - | - | - | - | - | 1.3526±0.0738^Ec^ | 2.3345±0.0413^Eb^ | - | - | - | 2.9895±0.0388^Ea^ | - | - | - | - | - |
| 12 | 5-Caffeoylquinic acid | 11.3100±0.1281^Bd^ | 10.2824±0.2113^Be^ | 7.3872±0.2403^Bf^ | - | - | - | - | - | - | - | - | - | 29.1051±0.3081^Ba^ | 23.1805±0.5308^Bb^ | 15.6474±0.3831^Bc^ | - |
| 15 | Caffeic acid | - | - | - | - | - | - | - | 15.0011±0.2617^Bb^ | - | - | - | 16.7421±0.2342^Ca^ | - | - | - | - |
| 17 | Coumaroylquinic acid | - | - | - | - | 0.1906±0.0007^Fa^ | 0.1066±0.0003^Gb^ | 0.1510±0.0007^Gc^ |  | 0.0917±0.0003^Fd^ | 0.0556±0.0002^Ff^ | 0.0772±0.0004^Ge^ | - | - | - | - | - |
| 19 | Vanillic acid hexoside | - | - | - | - | 0.8238±0.0031^Ea^ | 0.6656±0.0021^Fc^ | 0.4879±0.0022^Ff^ |  | 0.8177±0.0028^Eb^ | 0.6440±0.0020^Ed^ | 0.5941±0.0030^Fe^ | - | - | - | - | - |
| 21 | Vanillic acid-4-glucoside | 0.4328±0.0038^Cd^ | 0.1544±0.0008^De^ | 0.1368±0.0026^Ef^ |  | - | - | - | - | - | - | - | - | 1.1305±0.0058^Da^ | 0.8601±0.0048^Db^ | 0.5349±0.0108^Ec^ | - |
| 22 | Protocatechuic acid | - | - | - | - | - | - | - | - | 0.0807±0.0003^Fd^ | 0.2570±0.0008^Fc^ | 0.5173±0.0026^Fb^ | 0.7883±0.0110^Ea^ | - | - | - | - |
| 24 | Coumaric acid | - | - | - | - | - | - | - | 1.0755±0.0188^E^ | - | - | - | - | - | - | - | - |
| 27 | Protocatechuic acid | - | - | - | 1.5765±0.0454^Cb^ | - | - | - | - | - | - | - | - | - | - | - | 4.4000±0.1604^Ca^ |
| 29 | Sinapoyl-feruloyl-diglucoside | - | - | - | 0.5140±0.0148^Ed^ | - | - | - | 1.1005±0.0192^Eb^ | - | - | - | 1.0082±0.0141^Ec^ | - | - | - | 1.3186±0.0481^Da^ |
| **Flavonols and derivatives** | | | | | | | | | | | | | | | | | |
| 3 | Dihydrokaempferol | 12.1992±1.0677^Bi^ | 16.5965±1.1138^Bh^ | 15.5920±0.5674^Bh^ | 7.4943±0.5079^Bj^ | 33.0365±2.2870^Bcde^ | 33.2495±1.9962^Bcd^ | 58.6236±4.0439^Ba^ | 32.8280±0.6432^Bcde^ | 30.4501±1.9707^Bdef^ | 29.9488±0.5622^Bef^ | 53.2254±2.8365^Bb^ | 29.4417±0.6191^Bf^ | 28.2734±0.5957^Bf^ | 22.1654±0.9966^Bg^ | 35.8828±2.0410^Bc^ | 29.2373±0.6592^Bf^ |
| 4 | Kaempferol derivative | 60.3750±5.2844^Afg^ | 56.5562±3.7956^Ag^ | 44.1037±1.6051^Ah^ | 13.8022±0.4580^Ai^ | 123.6614±8.5606^Ab^ | 109.3233±6.5636^Ac^ | 115.5262±7.9691^Abc^ | 66.2811±0.8250^Af^ | 115.6378±7.4838^Abc^ | 112.5128±2.1122^Ac^ | 112.1259±5.9735^Ac^ | 51.70562±0.6975^Agh^ | 132.1186±2.7835^Aa^ | 97.1588±4.3685^Ad^ | 83.9398±4.7745^Ae^ | 52.5935±1.1441^Ag^ |
| 23 | Quercetin-hexoside-pentoside | - | - | - | - | - | - | - | - | 1.8086±0.1170^CDb^ | 1.1498±0.0216^Db^ | 1.5986±0.0852^Db^ | 11.6422±0.6712^Ca^ | - | - | - | - |
| 26 | Quercetin-3-O-rutinoside | 10.7127±0.9376^Bb^ | 10.1881±0.6837^Cb^ | 10.6593±0.3879^Cb^ | - | 8.8048±0.6095^Cc^ | 8.0503±0.4833^Cc^ | 6.7845±0.4680^Cd^ | - | 8.0141±0.5187^Cc^ | 4.3236±0.0812^Ce^ | 8.3959±0.4474^Cc^ | - | 13.8146±0.2910^Ca^ | 14.1828±0.6377^Ca^ | 10.0355±0.5708^Cb^ | - |
| 33 | Kaempferol-3-O-rutinoside | - | - | - | - | 1.2479±0.0864^Cb^ | 2.2732±0.1365^Ca^ | 2.2554±0.1556^Ca^ | 1.1827±0.0547^Cb^ | 1.2017±0.0778^Db^ | 1.0996±0.0206^Db^ | 2.1095±0.1124^Da^ | 1.1262±0.0402^Db^ | - | - | - | - |
| **Flavanones** | | | | | | | | | | | | | | | | | |
| 11 | Plantagoside | - | - | - | - | 7.8227±0.2915^a^ | 7.3743±0.2191^b^ | 5.3415±0.2895^e^ | 0.4192±0.0322^g^ | 7.0662±0.1011^b^ | 6.2245±0.0965^c^ | 5.8273±0.2206^d^ | 2.3048±0.2198^f^ | - | - | - | - |
| 14 | Eriodictyol-O-dihexoside | - | 1.4138±0.0281^b^ | 1.2385±0.0357^c^ | - | - | - | - | - | - | - | - | - | 0.7102±0.0294^Bd^ | 0.6639±0.0373^d^ | 4.4469±0.1927^a^ | - |
| 18 | Dimethoxylflavanone derivative | - | - | - | 8.0095±0.2229^b^ | - | - | - | - | - | - | - | - | 1.6559±0.0476^Ac^ | - | - | 37.5160±0.4041^a^ |
| **Flavone** | | | | | | | | | | | | | | | | | |
| 13 | Luteolin-4′-O-glucoside | - | - | - | - | 4.8139±0.2965^b^ | 4.4733±0.3617^b^ | 4.0333±0.2788^b^ | 47.8769±2.1144^a^ | - | - | - | - | - | - | - | - |
| **Flavanonol** | | | | | | | | | | | | | | | | | |
| 16 | Taxifolin-3-O-hexoside | 3.6695±0.1787^c^ | 2.9346±0.0998^d^ | 1.7531±0.1139^e^ | 0.1006±0.0134^f^ | - | - | - | - | - | - | - | - | 6.5691±0.2378^a^ | 5.3733±0.1956^b^ | 2.7526±0.1858^d^ | 1.6621±0.3120^e^ |
| **Flavan-3-ol** | | | | | | | | | | | | | | | | | |
| 25 | Epicatechin gallate | 1.9807±0.1516^e^ | 1.9124±0.1315^e^ | 1.7074±0.1084^ef^ | 0.6440±0.0905^h^ | 1.5239±0.0958f^g^ | 1.3496±0.0555^g^ | 1.2577±0.0532^g^ | - | 1.5237±0.0858^fg^ | 1.3147±0.0637^g^ | 1.2263±0.0849^g^ | - | 4.5487±0.2955^a^ | 4.2213±0.4147^b^ | 2.9203±0.2771^c^ | 2.5318±0.1251^d^ |
| **Other compounds (mAU)** | | | | | | | | | | | | | | | | | |
| 6 | Saccharide | 3737.8199 | 10482.1883 | 8600.3968 | 14181.1776 | 9646.2208 | 8986.3286 | 8284.4095 | 4260.5712 | 6238.6855 | 5380.3303 | 3137.5635 | 2211.7482 | 10059.9270 | 5031.6959 | 16240.3898 | 27824.3148 |
| 7 | Phloretin-2'-O-glucoside | 32069.8860 | 26715.5055 | 6693.4688 | 1062.4579 | 10899.5323 | 7459.5334 | 5963.3216 | - | 12153.4208 | 4353.5430 | 8402.8709 | - | 41042.2121 | 37933.6127 | 32213.9047 | - |
| 8 | Cis-ε-viniferin | 10417.9049 | 6953.5020 | 2274.7460 | 137.4697 | - | - | - | - | 13249.2260 | 4276.9176 | 10687.5986 | - | 7360.6097 | 7081.7904 | 3453.5861 | - |
| 20 | Gentiabavaroside | 4685.6893 | 4132.1565 | 2391.8800 | - | - | - | - | - | - | - | - | - | 6541.2225 | 4459.4225 | 3245.2827 | - |
| 28 | Restrisol A | 3601.1790 | 3304.6105 | 2951.4676 | - | 7274.3527 | 6149.4139 | 5989.5671 | - | 6663.5970 | 5845.2563 | 5379.6513 | - | 6301.2233 | 6797.1180 | 4813.5000 | - |
| 30 | Unknown | - | - | - | 68546.9514 | - | - | - | 164320.1693 | - | - | - | 144280.5419 | - | - | - | 129428.1985 |
| 31 | Benzyl alcohol-hexoside- pentoside I | 2952.6569 | 639.3203 | 1596.6688 | 733.5082 | 2945.2293 | 2466.3500 | 2596.3734 | 2732.0043 | 2754.7090 | 2657.5882 | 2462.9651 | 2558.1210 | 1293.0565 | 1136.8333 | 1263.6096 | 637.9050 |
| 32 | Unknown | 534.6651 | 192.8410 | 175.9261 | - | - | - | - | - | - | - | - | - | - | - | 185.9797 | - |
